# Anodal Transcranial Direct Current Stimulation Could Modulate Cortical Excitability and the Central Cholinergic System in Akinetic Rigid-Type Parkinson's Disease: Pilot Study

**DOI:** 10.3389/fneur.2022.830976

**Published:** 2022-03-24

**Authors:** Eungseok Oh, Jinse Park, Jinyoung Youn, Wooyoung Jang

**Affiliations:** ^1^Department of Neurology, Chungnam National University Hospital, Chungnam National University College of Medicine, Daejeon, South Korea; ^2^Department of Neurology, Haeundae Paik Hospital, Inje University, Busan, South Korea; ^3^Department of Neurology, Samsung Medical Center, Seoul, South Korea; ^4^Department of Neurology, Gangneung Asan Hospital, University of Ulsan College of Medicine, Gangneung, South Korea

**Keywords:** Parkinson's disease, tDCS, acetylcholine, short latency afferent inhibition (SAI), non-motor symptoms

## Abstract

**Background:**

Transcranial direct current stimulation (tDCS) is a non-invasive technique that has been widely studied as an alternative treatment for Parkinson's disease (PD). However, its clinical benefit remains unclear. In this study, we aimed to investigate the effect of tDCS on the central cholinergic system and cortical excitability in mainly akinetic rigid-type patients with PD.

**Methods:**

In total, 18 patients with PD were prospectively enrolled and underwent 5 sessions of anodal tDCS on the M1 area, which is on the contralateral side of the dominant hand. We excluded patients with PD who had evident resting tremor of the hand to reduce the artifact of electrophysiologic findings. We compared clinical scales reflecting motor, cognitive, and mood symptoms between pre- and post-tDCS. Additionally, we investigated the changes in electrophysiologic parameters, such as short latency afferent inhibition (SAI) (%), which reflects the central cholinergic system.

**Results:**

The United Parkinson's Disease Rating Scale Part 3 (UPDRS-III), the Korean-Montreal Cognitive Assessment (MoCA-K), and Beck Depression Inventory (BDI) scores were significantly improved after anodal tDCS (*p* < 0.01, *p* < 0.01, and *p* < 0.01). Moreover, motor evoked potential amplitude ratio (MEPAR) (%) and integrated SAI showed significant improvement after tDCS (*p* < 0.01 and *p* < 0.01). The mean values of the change in integrated SAI (%) were significantly correlated with the changes in UPDRS-III scores; however, the MoCA-K and BDI scores did not show differences.

**Conclusions:**

Anodal tDCS could influence the central cholinergic system, such as frontal cortical excitability and depression in PD. This mechanism could underlie the clinical benefit of tDCS in patients with PD.

## Introduction

The basic strategy of pharmacologic treatment for Parkinson's disease (PD) is focused on dopamine replacement, which is efficient for improving bradykinesia and rigidity (1–3). However, as the disease progresses, the pharmacologic response becomes less effective, and long-term treatment can result in motor fluctuations, such as levodopa-induced dyskinesia (1–3). Furthermore, many debilitating non-motor symptoms are observed, such as cognitive and behavioral deficits (4). In this context, deep brain stimulation (DBS) has been introduced, and it shows a therapeutic effect in patients with PD; however, DBS requires discrete patient selection because of surgical complications and some refractory symptoms (1, 5). Therefore, non-invasive, pharmacologically alternative treatment options are still required.

Among various non-invasive brain stimulation techniques, transcranial direct current stimulation (tDCS) has many advantages, such as low cost, minimal side effects, and easy application (6). The tDCS technique delivers a low-intensity current (1–2 mA) *via* a surface electrode placed on the scalp, enabling the neuronal resting membrane potential to possibly be modulated, thus altering cortical excitability (7–11). Therefore, tDCS has been widely studied for its efficacy in various neurodegenerative disorders (12). Many studies have shown the beneficial effects of tDCS on various symptoms in PD (9, 13–19). Ishikuro et al. reported that anodal tDCS in the frontal polar area improved the motor and executive function of patients with PD, and some meta-analytic studies have indicated that tDCS could have a positive effect on locomotive and gait function in PD, despite insufficient sample power to draw firm conclusions in most studies (15, 18, 20).

Although the mechanism of tDCS has been repeatedly studied, it is generally accepted that changes in cortical excitability are dependent on the polarity of the active electrode, current density, and total current stimulation charge. The change in cortical excitability can be assessed by estimating the amplitude of the motor evoked potential (MEP) on the contralateral target hand muscles (21–24). Generally, anodal tDCS increases cortical excitability, which could be reflected by an increase in MEP amplitude, whereas the inverse has been observed for cathodal tDCS (21, 23). However, many recent studies have revealed various mechanisms of tDCS beyond cortical excitability. We previously reported that anodal tDCS could affect biological processes associated with neuroprotection, and Scelzo et al. reported that enhanced cortical cholinergic activity, such as short latency afferent inhibition (SAI), was observed after tDCS (20, 25, 26).

Short latency afferent inhibition can be measured as a modulation of corticomotor output in response to afferent sensory input. MEP amplitude after transcranial magnetic stimulation (TMS) of M1 is suppressed by the sensory stimulation of the median nerve with a short time interval of 2–3 ms, and this phenomenon is believed to reflect inhibitory modulation by the central cholinergic circuit (27, 28). SAI is reduced in many types of dementia, and it is known for featuring cholinergic dysfunction (29, 30). Furthermore, many symptoms of PD, such as cognitive dysfunction, rapid eye movement (REM) sleep behavior disorders, visual hallucinations, and gait problems, have been reported to be associated with SAI (31–34). In addition, our group revealed that olfactory dysfunction and dysphagia in PD could be associated with reduced SAI, reflecting central cholinergic dysfunction (35, 36). Therefore, SAI is considered as a useful tool for assessing the integrity of central cholinergic interneurons non-invasively.

Considering that anodal tDCS could improve cognitive and gait problems in patients with PD, anodal tDCS could be hypothesized to modulate the central cholinergic activity estimated by SAI parameters. Therefore, we aimed to investigate whether anodal tDCS could improve motor, cognitive, and mood symptoms in patients with PD. Then, we evaluated electrophysiologic parameters, such as SAI, before and after anodal tDCS on M1 of the motor cortex.

## Patients and Methods

### Patients and Clinical Assessment

We prospectively enrolled 18 patients with PD in the Movement Disorder Clinic of Gangneung Asan, Gangwon-do, South Korea from January to December 2020. All of the patients with PD were diagnosed according to the UK Parkinson's Disease Society Brain Bank Diagnostic criteria. The study was approved by the ethical committee of Gangneung Asan Hospital, and written informed consent was obtained from all of the participants in accordance with the Declaration of Helsinki.

The exclusion criteria included the following: (1) a history of metallic implants, pacemaker, or skin/skull defects; (2) subjects with a history of seizure, intracranial hemorrhage, and brain neurosurgery; (3) participants treated with cholinergic or r-aminobutyric acid (GABA)ergic medication, such as acetylcholinesterase inhibitors, anticholinergics, benzodiazepines, and gabapentin; (4) atypical parkinsonism, such as progressive supranuclear palsy, multiple system atrophy, corticobasal syndrome, and dementia with Lewy bodies; (5) participants suspected of possible secondary parkinsonism, such as vascular Parkinsonism, with metabolic or toxic causes; (6) a history of medical or neurological diseases that could influence electrophysiologic studies, such as polyneuropathy and spinal cord injury; and (7) mean scores of tremor >1.5 during the “medication-on” state (sum of United Parkinson's Disease Rating Scale Part 3 (UPDRS-III) Items 20 and 21 divided by 7).

### Experimental Design

Before starting anodal tDCS application, basic demographic data, such as sex, age, disease duration, type of parkinsonism, and levodopa equivalent daily dose (LEDD), were assessed. The severity of motor symptoms was evaluated with UPDRS-III and Hoen and Yahr (H&Y) scales. Each subscore of tremor, rigidity, bradykinesia, and axial symptoms from the UPDRS-III was calculated as the sum of each relevant item (tremor: Items 20 and 21; rigidity: Item 22; bradykinesia: Items 23–26 and 31; and axial symptoms: Items 27–30). Cognitive function and mood were also evaluated with the Korean-Montreal Cognitive Assessment (MoCA-K) and Beck Depression Inventory (BDI).

Electrophysiologic parameters, such as resting motor threshold (RMT), MEP, central motor conduction time (CMCT), compound motor action potential (CMAP), and SAI by TMS, were recorded 1 h before anodal tDCS. Anodal tDCS was applied once per day for 5 consecutive days in a randomized selection. Then, 1 h after the final session, the UPDRS-III, MoCA-K, and BDI scores and the same electrophysiological parameters were re-evaluated by the same protocol.

### Intervention Protocol

Direct current was applied by a battery-driven, constant electrical stimulator (NeuroConn Ilmenau, Germany) through saline-soaked spongy electrodes (35 cm^2^). A motor hot-spot corresponding to the abductor pollicis brevis (APB) muscle of the dominant hand (compatible with C3 or C4 in a 10-to 200-month EEG system) was selected on the hemisphere obtained by TMS (anode), and the reference area was set to the contralateral frontopolar cortex. To minimize the interventional variance, before each session of tDCS application, the motor spot (M1) was re-estimated by TMS, and the center of the spongy electrode was located on the motor spot. The intensity of the electrical current was 2 mA for 20 min, and the current density was 0.057 mA/cm^2^. The motor hot-spot was defined as the TMS stimulation site in the contralateral hemisphere toward the affected APB, which could elicit the largest APB-MEPs, and the ramping period was 5 s from the beginning to the end of the session. There was no discomfort or adverse events in any subjects during the whole experiment.

### Conventional TMS Study

Conventional TMS parameters were investigated by a Magstim magnetic stimulator (Magstim Company, Dyfed, UK) using a figure eight coil. All of the participants were comfortably seated in a chair with armrests, and the surface Ag/AgCl electrode was applied over the APB muscle. First, the CMAP was recorded from the ipsilateral APB muscle with a supramaximal stimulus on the median nerve at the wrist. The lowest stimulation intensity that yielded a MEP of 50 μV peak-to-peak amplitude from the APB of more than 5 of 10 trials was defined as the resting motor threshold (rMT). To acquire maximal cortical MEP, the figure eight coil was tangentially located over the fronto-parietal area to stimulate the APB with handling to point backward and laterally at about a 45 degrees angle from the mid-sagittal axis. When the stimulus intensity (maximum intensity: 130% of RMT) was greater than the threshold for obtaining maximal MEP amplitude, the onset latency of the MEP was estimated. The onset latency was determined as the shortest latency from the MEP. The optimal sites to elicit maximal MEP were defined as motor hot spots when rMT was assessed. Next, the MEP amplitude ratio (MEPAR) was calculated as a ratio of the baseline-to-peak CMAP amplitude to the peak-to-peak amplitude of the MEP. Subsequently, the CMCT was calculated by the difference in latency from the cortical and cervical root evoked potentials.

### Short Latency Afferent Inhibition 1

The protocol for evaluating SAI followed the methods of Tokimura et al. (27). First, MEP without peripheral nerve stimulation was obtained as an unconditioned, control MEP. Next, conditioned MEP was investigated by conditioned stimuli delivered to the median nerve preceding cortical TMS with various interstimulus intervals (ISIs). ISIs were determined relative to the N20 latency obtained by somatosensory evoked potential (SEP). Two hundred sweep signals were averaged to identify the latency of N20. Five interstimulus intervals were used to evaluate the SAI (N20, N20 + 1 ms, N20 + 2 ms, N20 + 3 ms, and N20 + 4 ms), and twelve cortical stimuli after median nerve stimuli at the wrist were performed at each ISI. The peak-to-peak conditioned MEPs were averaged at each interval, and SAIs were expressed as the percentage of the control MEPs and conditioned responses at five ISIs. These five SAIs were averaged to obtain the grand mean as the integrated SAI. Visual-audio feedback on EMG monitoring was obtained for all of the subjects to maintain maximal relaxation during investigation. All of the SEP and MEP signals were amplified and filtered (bandwidth 3 Hz−3 kHz). Data were stored at a 10,000-Hz sampling rate for later analysis.

### Statistical Analysis

All of the data were analyzed with the commercial statistical software program GraphPad Prism, version 8.0 (GraphPad Software, Inc., San Diego, CA, USA). Wilcoxon's paired test was adopted to compare each variable between pre- and post-anodal tDCS application. The correlations between the change in SAI value (between pre- and post-tDCS) and changes in UPDRS-III, MoCA-K, and BDI scores (between pre- and post-tDCS) were explored by Pearson's linear coefficient value. All of the data are expressed as the mean and SD, and *p* < 0.05 was considered as statistically significant.

## Results

The demographic and baseline characteristics of each participant are presented in Table 1. There were 10 men and 8 women, and the mean age was 69.50 ± 7.16 years old. For the motor subtype of PD, 1 subject with the tremor dominant type, 2 with the intermediate type, and 15 with the akinetic-rigid type were enrolled. The SAI value was highly influenced by muscle contraction and tremor, so we excluded subjects with a high average tremor score through exclusion criteria. The mean BDI and MoCA-K scores were 18.22 ± 11.74 and 20.11 ± 6.64, respectively, indicating the burden of depression and cognitive impairment in patients with PD.

**Table 1:** Baseline demographic and clinical parameters of the enrolled PD subjects before/after anodal tDCS application.

	**PD subjects (pre-tDCS)** **(*n* = 18)**	**Post-tDCS**	***p*-value**
Age	69.50 ± 7.16		
Gender (male/female)	10/8		
Disease duration (months)	33.78 ± 12.71		
UPDRS-III	37.44 ± 12.12	32.11 ± 13.74	<0.01
Modified H and Y	2.27 ± 0.58		
LEDD	985.56 ± 214.95		
Type of PD (TD/intermediate/AR)	1/2/15		
Beck Depression Scale	18.22 ± 11.74	15.56 ± 10.98	<0.01
MoCA-K	20.11 ± 6.64	22.06 ± 6.53	<0.01

*These values represent the mean with the SD or the number of patients. PD, Parkinson's disease; tDCS, transcranial direct current stimulation; UPDRS-III, United Parkinson's Disease Rating Scale Part 3; H and Y, Hoeh and Yahr stage; MoCA-K, Korean version of Montreal Cognitive Assessment; TD: tremor-dominant; AR, akinetic rigidity*.

**Figure 1:**
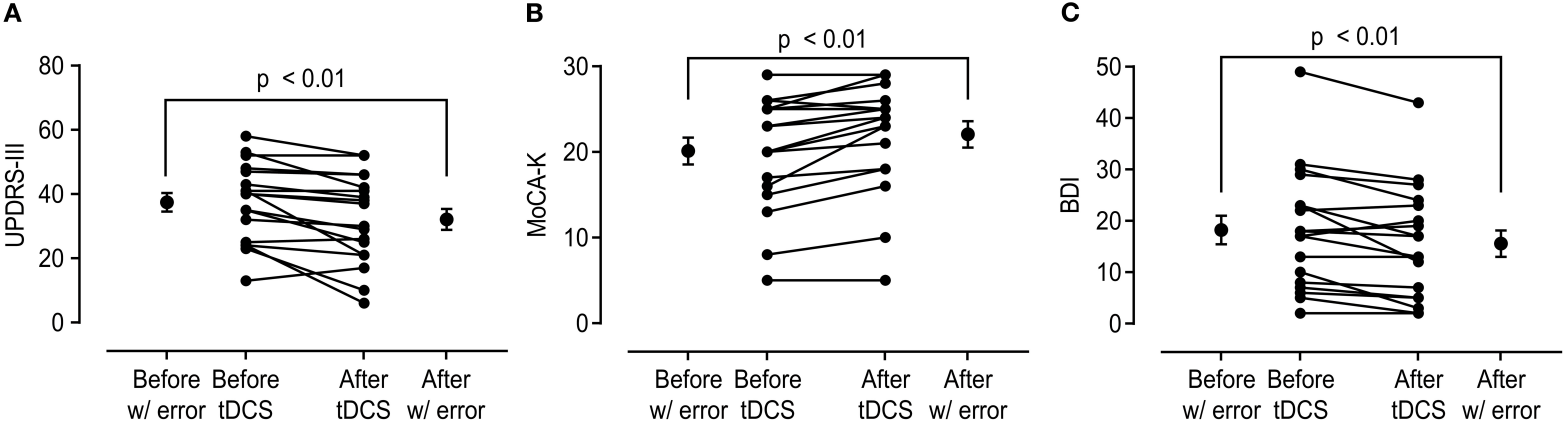
Change in the United Parkinson's Disease Rating Scale Part 3 (UPDRS-III), Korean version of Montreal Cognitive Assessment (MoCA-K), and Beck Depression Inventory (BDI) scores before and after anodal transcranial direct current stimulation (tDCS) application. **(A)** Total UPDRS-III score showed significant improvement after anodal tDCS (37.44 ± 12.12 vs. 32.11 ± 13.74, *p* < 0.01). **(B,C)** MoCA-K and BDI scores also showed significant changes in comparison between before and after tDCS (MoCA-K: 20.11 ± 6.64 vs. 22.06 ± 6.53, *p* < 0.01; BDI: 18.22 ± 11.74 vs. 15.56 ± 10.98, *p* < 0.01).

After anodal tDCS, UPDRS-III score, MoCA-K, and BDI showed significant improvement compared with those estimated in pre-tDCS status (Figure 1). The mean change in the UPDRS-III score was 5.33 ± 6.59 (*p* < 0.01), and the BDI and MoCA-K scores were 2.67 ± 3.48 (*p* < 0.01) and 1.94 ± 1.89 (*p* < 0.01), respectively. In the analysis of subscores of the UPDRS-III, the rigidity and bradykinesia scores were significantly improved after anodal tDCS (*p* < 0.01 and *p* < 0.05, Table 2).

**Table 2:** Comparison of mean UPDRS-III subscores in total PD subjects before and after tDCS application.

	**Pre-tDCS** **(mean ±SD)**	**Post-tDCS** **(mean ±SD)**	***p*-value**
UPDRS-III subscore			
Tremor score	4.72 ± 1.93	4.55 ± 2.46	0.74
Rigidity score	6.56 ± 3.38	4.33 ± 2.57	<0.01
Bradykinesia score	13.5 ± 5.92	11.78 ± 6.45	<0.05
Axial symptom score	7.72 ± 3.64	7.17 ± 4.02	0.13

*These values represent the mean with the SD of total patients. PD, Parkinson's disease; UPDRS-III, United Parkinson's Disease Rating Scale Part 3; tDCS, transcranial direct current stimulation*.

**Figure 2:**
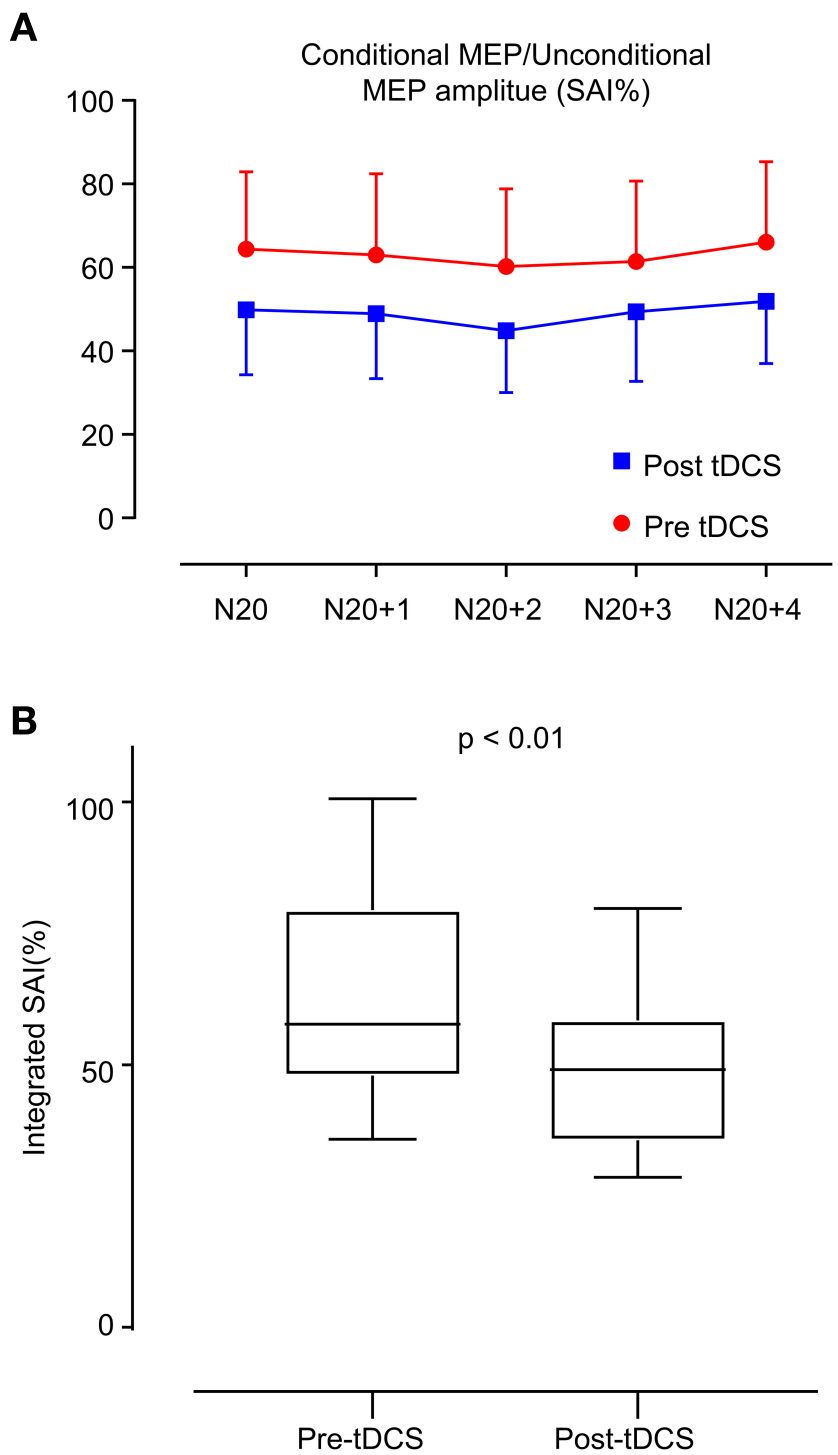
Change in short latency afferent inhibition (SAI). **(A)** SAI was evaluated in two ways: the red line indicates the estimation of SAI before anodal tDCS, and the blue line indicates the estimation after anodal tDCS. The interstimulus interval is 1 ms from N20 to N24. **(B)** Box plot of integrated SAI (%) distribution before and after anodal tDCS. After anodal tDCS, the SAI (%) showed a significant decrease compared with that before anodal tDCS (*p* < 0.01).

Table 3 shows the changes in electrophysiological parameters after anodal tDCS. The RMT, CMCT, and N20 latency did not significantly change between pre- and post-tDCS. However, MEPAR (%) and SAI response in all interstimulus intervals (N20, N20+ 1 ms, N20+ 2 ms, N20+ 3 ms, and N20+ 4 ms) revealed significant changes. The integrated SAI response post-tDCS showed a higher value than pre-tDCS (63.03 ± 18.77 vs. 48.99 ± 15.16, *p* < 0.01). Figure 2 shows a comparison of each interstimulus interval of the SAI and integrated SAI response before and after tDCS application.

**Table 3:** Change in electrophysiological parameters obtained by a conventional transcranial magnetic stimulation (TMS) study and SAI evaluation after tDCS.

	**Pre-tDCS**	**Post-tDCS**	***p*-value**
RMT (%)	79.16 ± 7.12	76.11 ± 6.98	NS
CMCT (ms)	7.93 ± 1.21	7.71 ± 1.23	NS
MEPAR (%)	45.27 ± 15.99	57.77 ± 28.34	<0.01
N20 (ms)	19.29 ± 1.27	18.94 ± 1.08	NS
SAI (%): N20	64.37 ± 18.59	49.91 ± 15.80	<0.01
SAI (%): N20 + 1 ms	63.04 ± 10.41	48.94 ± 15.57	<0.01
SAI (%): N20 + 2 ms	60.21 ± 18.60	44.86 ± 14.76	<0.01
SAI (%): N20 + 3 ms	61.46 ± 19.27	49.36 ± 16.59	<0.01
SAI (%): N20 + 4 ms	66.07 ± 19.22	51.88 ± 14.93	<0.01
Integrated SAI (%)	63.03 ± 18.77	48.99 ± 15.16	<0.01

*These values represent the mean with the SD. tDCS: transcranial direct current stimulation, PD, Parkinson's disease; RMT, resting motor threshold; CMCT, central motor conduction time; MEPAR, motor evoked potential amplitude ratio; SAI, short latency afferent inhibition*.

**Figure 3:**
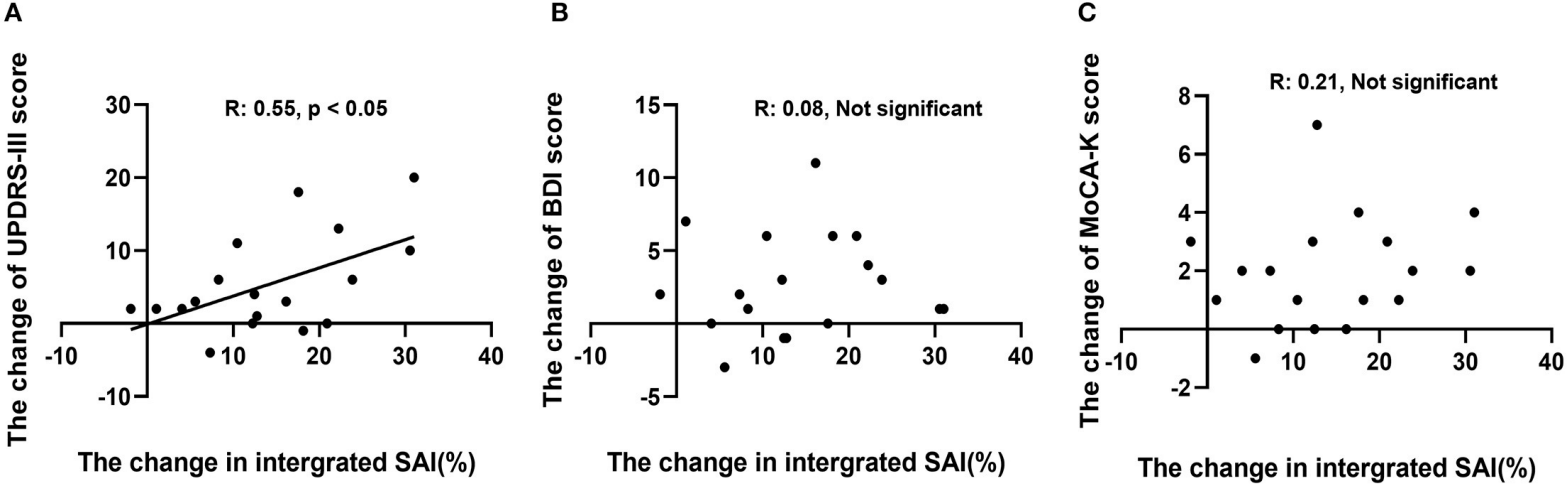
Correlation analysis between change in SAI (%) and change in each outcome variable. **(A)** Changes in UPDRS-III scores showed a positive, significant correlation (*R*: 0.55, *p* < 0.05). **(B,C)** Changes in MoCA-K and BDI scores did not reveal a significant correlation with SAI (%) changes.

A correlation analysis between the change in integrated SAI and UPDRS-III score showed a significant, positive correlation (*R* = 0.55, *p* < 0.05, Figure 3A). However, the differences between the changes in the MoCA-K and BDI scores relative to changes in the integrated SAI response were not statistically significant (Figures 3B,C).

## Discussion

Two major findings of this study are that motor symptoms, such as bradykinesia and rigidity and non-motor symptoms, such as cognition and depression were improved after anodal tDCS. Moreover, anodal tDCS elicited more inhibition estimated by the change in SAI (%) in PD subjects, and this change showed a correlation with the improvement of motor symptoms. The tDCS technique is a well-known treatment option for depression; moreover, we assumed that frontal executive function and attention could be improved after tDCS. However, to our knowledge, this study is the first investigating the effect of tDCS on central cholinergic function, and we confirmed that anodal tDCS might influence the central cholinergic system.

Transcranial direct current stimulation can modulate the cortical interneuronal system and cholinergic system of the human brain. In a previous study of 12 healthy controls, anodal tDCS of the primary motor cortex (M1) increased the SAI without changing the RMT or MEP size after stimulation (25). In contrast, the cathodal tDCS of the primary somatosensory cortex (S1) decreased the inhibitory effects of SAI in thirteen healthy controls (37). As a mechanism of tDCS, the low electrical current causes depolarization (anodal tDCS) and hyperpolarization (cathodal tDCS) of the resting membrane potential under the cortex, which presents limited stimulation. However, a number of studies have shown that anodal tDCS elicits the facilitation effect of the intracortex to disinhibit intracortical inhibition and modulate transcallosal inhibition (25, 38, 39). Therefore, the tDCS effect could affect nearby subcortical neurons, not only the stimulation cortex, and anodal tDCS affects cortical inhibitory circuits and M1 excitability (40, 41). In an early tDCS study, anodal tDCS of the human motor cortex decreased intracortical inhibition and increased paired-pulse excitability (25). Moreover, Antal et al. reported an improvement of chronic pain after anodal tDCS over M1, and they found that SAI was also reduced (38). This result suggests that anodal tDCS decreases various types of pain by adjusting the level of intracortical inhibition (38). In addition to inhibition within the cortical hemisphere, tDCS also mediates transcallosal inhibition *via* inhibitory interneurons (21).

The cortical cholinergic system is also highly associated with a feed-forward mechanism of the interneuronal system (42). Rivastigmine, a cholinesterase inhibitor, inhibits the facilitatory effect (PAS25) induced by anodal tDCS, reflecting that acetylcholine selectively modulates human cortical plasticity (43). Acetylcholine enhances synapse-specific cortical excitability after anodal tDCS, and a similar effect is implemented by L-dopa because dopamine can reverse the cortical excitability caused by anodal tDCS (43). Similar functions of acetylcholine and dopamine in cortical plasticity were highly associated with the brain connectome (44).

A similar mechanism of DBS as an explanation for the tDCS effect was also proposed. DBS improves the motor symptoms of PD to restore beta oscillations in the subcortico-cortical functional network, and tDCS could also non-invasively modulate the cortical excitabilities and oscillatory components of the PD brain (10, 23, 39, 45).

Several studies have emphasized that motor symptoms, such as those affecting the isometric grip test and hand dexterity, were improved after anodal tDCS of the motor cortex (15, 46, 47). However, non-motor symptoms of PD, such as depression, fatigue, and frontal executive function, were improved after anodal tDCS on the frontal cortices (19). In addition to the stimulation site, stimulation intensity, duration, and polarity could differentially affect cortical excitability. For example, Shahid et al. reported that anodal tDCS for 20 min on M1 definitely increased MEP sizes compared with short durations of stimulation, such as 5 and 10 min (11). Additionally, cathodal tDCS reduces the SAI through intracortical inhibitory circuits, such as the GABAergic system and cholinergic system (37). Consider this evidence together, tDCS can modulate subcortico-cortical networks in the human brain by regulating synaptic plasticity and changing the levels of various neurotransmitters.

The changes in motor and non-motor symptoms of PD could also be associated with neurotransmitters in the cortico-cortical network. It was reported that the levels of dopamine, serotonin, GABA, N-methyl-D-aspartate (NMDA), and acetylcholine could be changed after anodal and cathodal tDCS (48). The SAI (%) might also be influenced by cholinergic, GABAergic, and dopaminergic pathways (41, 42). Furthermore, dopamine release could be increased in the ventral striatum by anodal tDCS to the dorsolateral prefrontal cortex (DLPFC), which was evaluated with [11C]-raclopride PET, and tDCS over the primary motor cortex (M1) improved motor learning through reward-based feedback, which might be associated with the dopaminergic pathway (46, 49). Therefore, the mechanism of the anodal tDCS effect might be associated with various neurotransmitters.

Another possible explanation of the anodal tDCS effect is connectivity between the motor cortex and adjacent brain structures. M1 pyramidal neurons are locally connected with the cortex, striatum, thalamus, and brainstem nuclei *via* intraencephalic neurons, pyramidal tract neurons, and cortico-thalamic neurons (50). Therefore, M1 stimulation changes the firing rate and synchronization of the midbrain to the M1 loop, which is associated with motor controls in PD. For this point, the modulation of cholinergic system by anodal tDCS could be addressed. The central cholinergic system, which is divided into the brain stem and basal forebrain cholinergic system, is highly connected to the thalamus and ventral tegmental area (51). Therefore, anodal tDCS over M1 influences the cortico-subcortical pathway linked with various neurotransmitters and induces changes in the motor and non-motor symptoms of PD. In this study, changes in SAI (%) were not associated with MoCA-K and BDI scores but with UPDRS-III scores, reflecting the contribution of the cholinergic system to motor symptoms in PD. One possible explanation is the connection between the cholinergic system and the basal ganglia. Classically, motor symptoms in PD are regarded as the consequences of the loss of dopaminergic neurons and the discordance of the striatal input system between the direct and indirect pathways (52). However, much evidence has shown that the striatal cholinergic system also plays a crucial role in the regulation of striatal activity, such as triggering of dopamine release, modulation of glutamate release from medium spiny neurons, and feedback inhibition *via* the muscarinic 4 receptor (53, 54). There are several lines of evidence that altered cholinergic tone in the striatum could contribute to the occurrence of motor symptoms in PD animal models (55, 56). Another possible explanation is that axial symptoms in PD, such as impaired gait, falls, and poor posture, could be associated with the degeneration of cholinergic nuclei, such as the pedunculopontine nucleus (PPN) (54, 57). Pienaar et al. reported that pharmacogenetic stimulation of the PPN could reverse motor symptoms in PD animal models, indicating that the functional modulation of the PPN is relevant to parkinsonian motor symptoms (58). In human studies, there have been many reports that cholinergic dysfunction could be relevant to gait disturbances or axial motor symptoms in Alzheimer's disease and PD (57, 59). Furthermore, SAI has also been reported to be associated with cognitive function, psychologic symptoms, and attention (60–62). Cognitive impairment and attentional deficits affect postural control through the integration of sensory input and motor planning in walking. Therefore, central cholinergic dysfunction could affect the deterioration of axial symptoms, such as impaired gait, postural instability, and freezing (63).

On the other hand, we did not discover the anodal tDCS effect for the axial symptoms in PD. There have been several studies showing the efficacy of tDCS on axial symptoms in PD (13, 18, 64, 65). Pol et al. suggested that anodal tDCS over the motor areas has revealed positive effect on gait in a meta-analysis (18). However, there have been contradictory results that anodal tDCS on the motor cortex did not show efficacy on gait. Dagan et al. reported that single motor area stimulation did not reveal the improvement of gait in PD, while combined stimulation with the prefrontal cortex led to a positive result (66–68). Furthermore, Lu et al. reported that bilateral anodal tDCS on M1 macrophages did not show efficacy in freezing gait in PD. These contradictory results might be caused by various factors, such as electrode size and type, stimulation density, and stimulation area. In this study, our stimulation protocols used relatively low intensity current and the unilateral dominant motor cortex, which is responsible for the hand muscles. Therefore, our stimulation methodology was concomitant with that in tDCS studies showing negative results on gait and other axial symptoms. Identifying the optimal target and method for tDCS on gait is required in future research.

This study has several limitations. First, we evaluated SAI in the “medication-on” state; accordingly, the “off” state SAI was not compared. In a previous study, SAI was normal in the PD “off” state but decreased on the more affected side in the PD “on” state (28). Therefore, the effect of dopaminergic medication on SAI must be more clearly clarified. Second, variable neurotransmitters could influence SAI, such as r-aminobutyric acid (GABA), as well as cholinergic activity (41, 42, 69). Udupa et al. reported inhibitory interactions of long interval cortical inhibition (LICI) and short interval cortical inhibition (SICI) with SAI, indicating the GABAergic modulation of central cholinergic activity (55, 70). Moreover, dopamine could modulate acetylcholine levels through dopaminergic receptors located on cholinergic neurons. The dopaminergic stimulation of the nucleus accumbens could modulate the excitability of cholinergic neurons in the basal forebrain through GABAergic projections (71, 72). Additionally, cholinergic excitatory projection could modulate dopamine transmission in the prefrontal cortex through close coordination with glutaminergic and GABAergic activity (72, 73). Therefore, considering that tDCS could increase dopamine levels in the striatum in animal models, its effect on SAI and clinical benefit in PD might be very complicated interactions of various neurotransmitter systems, and the possible contributions of other neurotransmitters to tDCS stimulation could not be confirmed in this study. Although we attempted to exclude this confounding condition, this point should be considered when interpreting our results. SICI and LICI reflecting GABAergic receptor activity could be evaluated in future investigations to more clearly confirm the tDCS effect on SAI with paired pulse TMS studies. Third, the follow-up of cognitive function was not evaluated by alternative forms of MoCA. Therefore, cognitive improvement could be a learning effect. Fourth, we excluded patients with PD with prominent tremor because evident tremor could influence electrophysiolgic findings, such as SAI (%) and TMS parameters. Thus, our results could not be generalized in tremor-dominant patients with PD. Fifth, the sample size of this study is very small, so it should be regarded as a preliminary or pilot study. A more sophisticated study design with a larger sample size and more intricate subtyping is necessary for the generalization of our results to all patients with PD. Finally, this study was an open-label pilot study without comparison with a sham stimulation group or a group with cathodal stimulation. Therefore, the clinical benefit of this study could be the placebo effect, and it is not clear whether the polarity of tDCS influences changes in SAI values. Furthermore, the long-term effect of tDCS on the SAI value was not investigated, so caution should be exercised when interpreting our data. Further studies with larger sample sizes and randomized, controlled sham, or cathodal stimulation groups are necessary to overcome this limitation.

In conclusion, we observed that SAI (%) could be modulated by anodal tDCS in patients with PD, in parallel with the clinical improvement of motor and non-motor symptoms associated with cholinergic dysfunction. To the best of our knowledge, this study is the first regarding anodal tDCS on SAI (%) in patients with PD. Our findings indicate that central cholinergic dysfunction could be modulated by anodal tDCS in PD, and further, larger clinical trials are warranted.

## Data Availability Statement

The raw data supporting the conclusions of this article will be made available by the authors, without undue reservation.

## Ethics Statement

The studies involving human participants were reviewed and approved by the Ethical Committee of Gangneung Asan Hospital. The patients/participants provided their written informed consent to participate in this study.

## Author Contributions

EO proposed the research idea, performed the data analysis and interpretation, and wrote the manuscript. JP and JY provided the clinical suggestions. WJ confirmed the conception and design of the research, wrote the manuscript, and prepared the manuscript for submission. All authors contributed to the article in meaningful manners, contributed to the article, and approved the submitted version.

## Funding

This research was financially sponsored by Samil Pharm. Co., Ltd. (2019-06-035-005) and the Gangneung Asan Hospital Biomedical Research Center promotion fund (2020IB005). The funders were not involved in the study design, collection, analysis, interpretation of data, writing of this article, or decision to submit it for publication.

## Conflict of Interest

The authors declare that the research was conducted in the absence of any commercial or financial relationships that could be construed as a potential conflict of interest.

## Publisher's Note

All claims expressed in this article are solely those of the authors and do not necessarily represent those of their affiliated organizations, or those of the publisher, the editors and the reviewers. Any product that may be evaluated in this article, or claim that may be made by its manufacturer, is not guaranteed or endorsed by the publisher.
